# Magnitude and associated factors of anemia among adolescent girls in Ethiopia: a systematic review and meta-analysis

**DOI:** 10.1186/s13690-022-00942-y

**Published:** 2022-08-11

**Authors:** Kidanemaryam Berhe, Freweini Gebrearegay, Hadush Gebreegziabher, Lemlem Weldegerima, Amaha Kahsay, Haven Hadush, Brhane Gebremariam, Berhane Fseha, Gebrehiwot Gebremariam, Natnael Etsay, Micheale Hailu

**Affiliations:** 1 Department of Nutrition, School of Public Health, College of Health Sciences, Mekelle University, Mekelle City, Tigray, Ethiopia; 2 Department of Pediatrics and Child Health Nursing, School of Nursing, College of Health Sciences, Mekelle University, Mekelle City, Tigray, Ethiopia; 3 Tigray Institute of Policy Studies, Mekelle City, Tigray, Ethiopia; 4 Department of Public Health, College of Health Sciences, Adigrat University, Adigrat City, Tigray, Ethiopia; 5 Department of Midwifery, College of Health Sciences, Adigrat University, Adigrat City, Tigray, Ethiopia; 6 Department of Biomedical Sciences, College of Health Sciences, Adigrat University, Adigrat City, Tigray, Ethiopia

**Keywords:** Anemia, Magnitude, Adolescent girls, Associated factors, Ethiopia

## Abstract

**Background:**

In Ethiopia, there are primary studies on adolescent anemia with imprecise and inconclusive findings. Besides, there was no meta-analysis pooled the magnitude and associated factors of anemia among adolescent girls in Ethiopia. Estimating the pooled magnitude and associated factors of anemia among adolescent girls is helpful for evidence-based interventions in Ethiopia.

**Methods:**

The authors used a preferred reporting item for systematic reviews and meta-analysis (PRISMA). We included articles and survey reports published until May 2021 using searching engines of Google, Google Scholar, PubMed, Scopus, and Cumulative Index to Nursing and Allied Health Literature. To assess the quality of studies, we used Newcastle–Ottawa quality assessment scale for non-randomized. Two authors independently assessed the quality of the studies. We computed the pool magnitude and odds ratio of the associated factors with their 95%CI using Comprehensive Meta-Analysis software. Publication bias assessed using funnel plots and Egger’s test.

**Result:**

In this review, we included a total of 15 studies with 9,669 adolescent girls. Using the random-effects model, the pooled magnitude of anemia among the Ethiopian adolescent girls was 19.1% (95%CI: 16.1%, 24.6%). The associated factors were attained menarche (adjusted odds ratio (AOR) = 1.96), ≥ 5 days of blood flow during menses (AOR = 6.21), food insecurity (AOR = 1.48), inadequate diet diversity score (AOR = 2.81), presence of intestinal parasite (AOR = 3.51), low body mass index (AOR = 2.49), and rural residence (AOR = 1.79).

**Conclusion:**

The pooled magnitude of anemia among adolescent girls in Ethiopia was 19.1% depicting a mild public health problem; while attained menarche, ≥ 5 days’ blood flow during menses, food insecurity, inadequate diet diversity score, intestinal parasites, low body mass index, and rural residence were the associated factors. Hence, addressing health and nutrition wellness of adolescent girls should be center of concern in health, nutrition, agriculture, research, strategies and policies in Ethiopia.

**Supplementary Information:**

The online version contains supplementary material available at 10.1186/s13690-022-00942-y.

## Background

Adolescence comprises the ages of 10–19 years which is a critical period during the life cycle. There are an estimated 1.8 billion adolescents in the world, with 90% living in low and middle-income countries. The rapid growth and development of adolescent are often referred to as the second window of opportunity to make a positive impact on nutrition, after the first 1,000 days of life. Good nutrition for adolescent girls impacts their current and future health, improves human capital, helps to break the intergenerational malnutrition cycle, ensures optimal maternal health and birth outcomes for themselves and their families now, and later in life laying the foundation for improved growth and development of the next generation [1, 2]. Insufficient emphasis has been given to adolescent girls’ nutrition including anemia, and they have been missed from global and local programs. Anemia is defined as low red blood cells or low concentration of hemoglobin which compromises their oxygen-carrying capacity and body’s physiologic needs. Based on the World Health Organization recommendation for non-pregnant adolescent girls, a hemoglobin concentration of 11–11.9 g/dl is mild anemia, 8–10.9 g/dl is moderate anemia, and < 8 g/dl is severe anemia and the hemoglobin cut-off points should be adjusted for an altitude of above 1,000 m above sea level and smoking status. This is because high altitude and smoking are known to increase hemoglobin concentration which may underestimate the magnitude of anemia if the standard anemia cut-offs are applied without adjustment. Public health significance of anemia in population is based on its magnitude; ≥ 40% (severe), 20–39.9% (moderate), and 5–19.9% (mild) [3]. Worldwide, the magnitude of anemia among adolescent girls ranged from 15 to 54% with the highest magnitude in Asia (54%) and African countries (35%) [4] but the World Health Assembly has been working to reduce anemia by 50% by 2025 [5]. The causes of anemia include iron deficiency, folate deficiency, vitamin B_12_ deficiency, vitamin A deficiency, acute and chronic inflammation, parasitic infection, and inherited or acquired disorders that affect hemoglobin synthesis, red blood cell production, or red blood cell survival [5, 6]. Evidence showed that anemia affects adolescents’ mental health [7], their educational performance, and cognition [8]. Different primary studies conducted on anemia among adolescent girls in Ethiopia came up with imprecise and inconclusive findings. Besides, there was no systematic review and meta-analysis that estimated the pool magnitude of anemia and its associated factors among adolescent girls in Ethiopia. Therefore, this systematic review and meta-analysis aimed to estimate the pool magnitude and associated factors of anemia among adolescent girls which is important for evidence-based interventions.

## Methods

### Eligibility criteria and information sources

In this systematic review and meta-analysis, we included studies conducted in Ethiopia with the objective of assessing the magnitude or prevalence of anemia and associated factors among adolescent girls which were published or reported in English. Studies were assessed for inclusion criteria using title, abstract, and a full review of the studies. In this review, we used a preferred reporting items for systematic review and meta-analysis (PRISMA 2020) checklist [9]. We included all eligible studies that were published or reported until May 30–2021 (with no lower time-bound) were included in this systematic review and meta-analysis. We used Mendeley Desktop 1.19.4 to facilitate the article selection process and manage citation.

### Search and study selection

In this systematic review and meta-analysis, we identified studies by searching electronic databases, scanning reference lists of articles. We used search engines of Google, Google Scholar, PubMed, Scopus, and Cumulative Index to Nursing and Allied Health Literature (CINAHL) to find out articles and survey reports. PubMed database is one of the most comprehensive sources of health studies in the world but its coverage is not complete. Thus, we considered additional search databases to make our searching comprehensive and complete. Searching activities were performed by two authors independently using the following search terms to find all relevant studies in the search databases: ‘magnitude of anemia’, ‘prevalence of anemia’, ‘assessment of anemia’,’anemia disorder’, ‘anemia’, ‘associated factors’, ‘risk factors’, ‘determinants’, ‘adolescent’, ‘adolescent girls’ and ‘Ethiopia’ separately and/or in combination using the Boolean operator like ‘OR’ or ‘AND’.

### Data collection process and data items

We used a predefined data extraction format to collect information. Name of the author(s), publication year, region, study design, community/school-based, age of the adolescents, sample size, and response rate were some of the information extracted for both the magnitude and associated factors. The magnitude, severity (if reported), and hemoglobin cut-offs of anemia were collected for the magnitude of anemia; and similarly, the name and adjusted odd ratio or *p*-value of the associated factors were collected.

### Assessment of quality of the studies

We assessed the quality of the studies using the criteria proposed in the protocol called the Newcastle–Ottawa quality assessment scale for non-randomized studies [10]. The parameters we used to assess the quality of the studies were: sampling strategy, inclusion/exclusion criteria, sample size, hemoglobin cut-offs for anemia, and statistical models used. A total score of 9 stars was considered as maximum and zero as a minimum. A study was considered a high quality if it scored 6 and above [11]. The quality of the studies was assessed by two authors independently.

### Summary measures

Magnitude of anemia among adolescent girls in Ethiopia was the primary outcome of this systematic review and meta-analysis. WHO defines anemia in adolescent as low blood hemoglobin concentration, below 12 g/dl or hematocrit level less than 36% [3]. Studies included in this systematic review and meta-analysis used the WHO cut-offs. In this meta-analysis, both blood sample sources i.e., capillary blood samples and venous blood samples were considered to measure hemoglobin concentration. The cyanmethemoglobin and the HemoCue system were applied to determine the population prevalence of anemia. These systems are methods generally recommended for determining the population prevalence of anemia [3, 12]. The second outcome of this systematic review and meta-analysis was the associated factors of anemia among adolescent girls. The effect sizes of this systematic review and meta-analysis were the magnitude of anemia among adolescent girls and the adjusted odds ratios (AORs) of the associated factors for anemia. All significant factors mentioned in the primary studies were stated in the systematic review and factor that was mentioned as statistically significant in at least two primary studies was included in the meta-analysis part. Accordingly, inadequate diet diversity score (consuming ≤ 3 food groups from 9 food groups [13]), food insecurity (low household food insecurity access scale [14]), low body mass index (BMI for age z-score < -2SD [15]), Attained menarche (starting menstruation cycle at least once), ≥ 5 days’ blood flow during menses [16], intestinal parasite (presence of parasite in the stool examination), and rural residence were include in the meta-analysis.

### Statistical methods and analysis

In this systematic review and meta-analysis, we used Comprehensive Meta-Analysis (CMA) software version 3.3.07 (November 20, 2014) for statistical analysis. Due to the heterogeneity nature of the studies, the random effect model was used as a method of analysis. Subgroup analysis by the level of study (community-based/facility-based/refuge camp) was performed for the magnitude of anemia among adolescents but no subgroup analysis by age, or regional states was performed due to a limited number of studies. Magnitude of anemia with its 95%CI was pooled. The factors associated with anemia were identified by looking at *p*-value, adjusted odds ratio (AOR) and its 95%CI in each primary study. A separate category of the meta-analysis was prepared to analyze the odds ratio of each associated factor. The data (number) for each category of the variable was entered to the CMA software and the adjusted odds ratio produced as an outcome of the software. The effect sizes were reported with their 95%CIs. Sensitivity analysis was used to examine effect of aberrant studies. The findings of the systematic review and meta-analysis are presented using texts, tables, and graphs.

### Publication bias and heterogeneity

In this systematic review and meta-analysis, publication bias was assessed using funnel plots and Egger’s test. In the funnel plot, the symmetry was assessed by visual examination, and Egger’s test. We used a *p*-value < 0.05 to declare the statistical significant of publication bias [17]. I^2^ was used to check the heterogeneity of studies included in this systematic review and meta-analysis. I^2^ test statistics of < 50%, 50–75%, and > 75% were declared as low, moderate, and high heterogeneity, respectively [18].

## Results

### Study selection

**Fig. 1:**
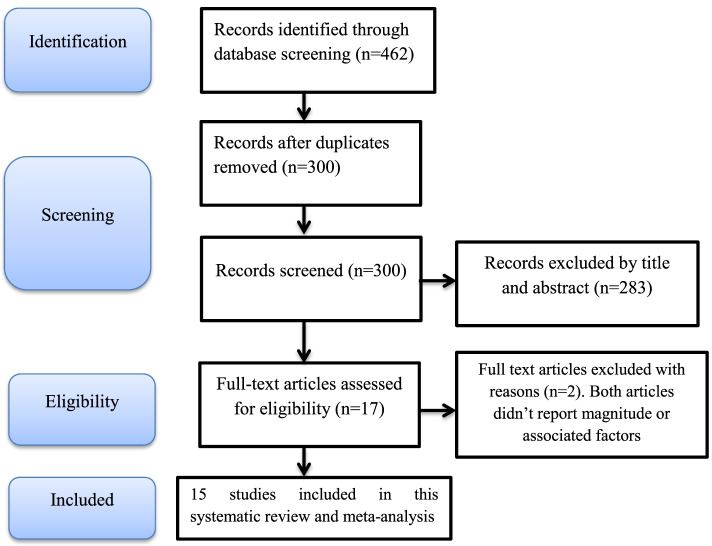
Flow diagram of the studies included in the systematic review and meta-analysis for magnitude and associated factors among adolescent girls in Ethiopia, 2021

In the initial search, we found a total of 462 records from different electronic search databases. We didn’t limit the lower time-bound to widen the comprehensiveness of the search but the upper time bound was until May 30, 2021. We removed 162 duplicate records. Likewise, 283 records were excluded from this analysis after screening the title and abstract of the studies. Finally, we left with 17 records. After assessing the full texts of the 17 records for their eligibility, 2 records [19, 20] were further excluded by the exclusion criteria. Finally, 15 studies [21–35] were found to be eligible for this systematic review and meta-analysis (Fig. 1).

### Characteristics of the studies

All the studies included in this systematic review and meta-analysis were cross-sectional studies. Eight studies were school-based, six studies were community-based, and one study was from a refugee camp. A total of 9,669 adolescent girls were included in this systematic review and meta-analysis. The sample size ranged from 177 [30] to 3,165 [24]. Out of the 15 studies included in this systematic review and meta-analysis, 5(33.3%) studies were conducted in South Nation, Nationalities, and Peoples (SNNP) of Ethiopia, 3(20%) studies were conducted in Oromia regional state, 2(13.3%) were conducted in Amhara regional states, 2(13.3%) studies were conducted at the national level (Ethiopia), 1(6.7%) study was conducted in the capital city of Ethiopia (Addis Abeba), 1(6.7%) study was conducted in Somali regional state, and 1(6.7%) study was conducted in Afar regional states. The studies included in this systematic review and meta-analysis showed that the magnitude of anemia among adolescent girls in Ethiopia ranged from 8.7% [32] to 32% [34]. All the studies included in this systematic review and meta-analysis have scored 7 and above (Quality score ≥ 7) (Table 1).

**Table 1:** Characteristics of studies included in the systematic review and meta-analysis for magnitude and associated factors of anemia among adolescent girls in Ethiopia, 2021

**S.no**	**Author (s) and Publication year**	**Region**	**Residence**	**Type of study**	**Age of the adolescent**	**Sample size**	**Response rate**	**Indicator**	**Magnitude of anemia (%)**				**Quality score (Out of 9)**
									**Total**	**Mild**	**Moderate**	**Severe**	
1	Regasa T et al. 2019 [31]	Oromya	Urban and rural	School based CS	Adolescent girls (10–19)	454	98	Hgb < 12 g/dl	27	4	23	0	9
2	Gebreyesus S et al. 2019 [33]	SNNP	Urban and rural	Community based CS	Adolescent girls (10–19)	1323	100	Hgb < 12 g/dl	29.2	25.2	3.8	0.3	9
3	Teji K et al. 2016 [34]	Oromya	Urban and rural	Community based CS	Adolescent girls (10–19)	600	91	Hgb < 12 g/dl	32	NR	NR	1.8	8
4	Engidaw M et al. 2018 [21]	Somali	Urban and rural	CS on refugee camp	Adolescent girls (10–19)	456	95.8	Hgb < 12 g/dl	22	NR	NR	NR	7
5	Tesfaye M et al. 2015 [28]	SNNP	Urban	School based CS	Adolescent girls (12–19)	238	100	Hgb < 12 g/dl	19.3	83.9	12.9	3.2	8
6	Shaka M et al. 2018 [30]	SNNP	Urban and rural	School based CS	Adolescent girls (10–19)	177	100	Hgb < 12 g/dl	18.1	NR	NR	NR	7
7	Teni M et al. 2017 [29]	SNNP	Urban and rural	School based CS	Adolescent girls (10–19)	442	96	Hgb < 12 g/dl	12	11.1	0.9	NR	9
8	EDHS 2016 [24]	National	Urban and rural	Community based CS	Adolescent girls (15–19)	3,165	100	Hgb < 12 g/dl	19.9	15.6	3.9	0.4	8
9	EPHI2016 [27]	National	Urban and rural	Community based CS	Adolescent girls (15–19)	313	100	Hgb < 12 g/dl	11.8	0	10.5	1.3	8
10	Seyoum Y et al. 2019 [32]	Oromya	Rural	Community based CS	Adolescent girls (15–19)	257	100	Hgb < 12 g/dl	8.7	7.9	0.8	0	7
11	Seid O et al. 2015 [25]	Afar	Urban and rural	School based CS	Adolescent girls (14–19)	338	100	Hgb < 12 g/dl	22.8	17	5	0.8	8
12	Mengistu G et al. 2019 [22]	Amhara	Urban and rural	School based CS	Adolescent girls (10–19)	443	95.5	Hgb < 12 g/dl	11.1	10.8	0.2	0	8
13	Gonete K et al. 2018 [23]	Amhara	Urban and rural	School based CS	Adolescent girls (15–19)	462	100	Hgb < 12 g/dl	25.5	23.6	1.5	0.4	9
14	Alemu T et al. 2020 [26]	SNNP	Rural	Community based CS	Adolescent girls (10–19)	407	98.2	Hgb < 12 g/dl	15.2	13.7	1.5	NR	9
15	Demelash S et al. 2019 [35]	Addis Ababa	Urban	School based CS	Adolescent girls (15–19)	594	95.9	Hgb < 12 g/dl	21.1	NR	NR	NR	8

*EDHS* Ethiopia Demographic and Health Survey, *EPHI* Ethiopia Public Health Institute, *SNNPR* South Nation, Nationalities, and Peoples, *CS* Cross-Sectional, *Hgb* Hemoglobin, *g.* gram, *dl* deciliter, *NR* Not Reported, Quality score is for assessment of risk of bias for each included study

Out of the fifteen studies included in this systematic review and meta-analysis, twelve studies identified associated factors for anemia among adolescent girls (Table 2).

**Table 2:** Summary of the associated factors from studies included in this systematic review and meta-analysis for magnitude and associated factors of anemia among adolescent girls in Ethiopia, 2021

s.no	Author (s) and publication year	Associated factors	Adjusted Odds Ratio (95%CI)
1	Regasa T et al. 2019 [31]	Late adolescent age (15–19)	3.8 (2.3, 8.5)
		Rural residence	3.4 (1.9,7)
		Attained menarche	2.3 (1.34,4.2)
2	Gebreyesus S et al. 2019 [33]	Early adolescent age (10–14)	1.98 (1.03, 3.82)
		Food insecure households	1.98 (1.03, 3.82)
		Poor/no knowledge about anemia	1.58[1.09,2.29]
3	Teji K et al. 2016 [34]	Late adolescent (15–19)	*P*-value = 0.001 (from chi-square test)
		Rural residence	*P*-value = 0.007(from chi-square test)
		Food insecure households	*P*-value = 0.02 (from chi-square test)
		Farmer occupation of the mother	*P*-value = 0.02 (from chi-square test)
4	Engidaw M et al. 2018 [21]	Late adolescent (15–19)	1.95 (1.09, 3.47)
		≤ once per week consumption of hem iron source foods	11.42 (3.42, 38.18)
5	Tesfaye M et al. 2015 [28]	Attained menarche	2.34 (1.20, 4.54)
		Daily worker father	2.86 (1.15, 7.09)
		≥ 5 family size	2.58 (1.11,5.96)
		Father illiteracy	9.03 (4.29,18.87)
		Presence of intestinal parasite	5.37(2.65,10.87)
		Low body mass index (BMI)	2.54 (1.17,5.51)
6	Shaka M et al. 2018 [30]	Early adolescent (10–14)	4.75 (1.69,13.35)
		Rural residence	4.37 (1.54,12.46)
		≥ 5 family size	9.82 (2.42,39.88)
7	Teni M et al. 2017 [29]	Early adolescent (10–14)	3.4 (1.4—8.2)
		Walking barefoot	2.7 (1.1, 6.6)
8	Seid O et al. 2015 [25]	≤ once per week consumption of hem iron source foods	2.03(1.13,3.01)
		≤ once per week milk consumption	2.2(1.2,4.3)
		≤ once per week vegetable consumption	2.4(1.24,4.67)
9	Mengistu G et al. 2019 [22]	≥ 5 days of blood flow during menses	2.4 (1.08,5.44)
		≥ 5 family size	3.2 (1.29, 7.89)
		Presence of intestinal parasite	2.7(1.19,6.21)
		Low body mass index (BMI)	3.2(1.43,7.05)
		Low income (≤ 500 Ethiopian Birr)	10 (2.49,41.26)
10	Gonete K et al. 2018 [23]	Food insecure households	1.9(1.1, 3.5)
		Living with either parents or guardians	2.2(1.08,4.6)
		Inadequate diet diversity	2.1(1.3,3.5)
11	Alemu T et al. 2020 [26]	≥ 5 days of blood flow during menses	6.4 (1.55, 27.0)
		≥ 5family size	0.37(0.16, 0.92)
		Inadequate diet diversity	3.6(1.7, 7.7)
		History of malaria infection	3.2(1.4, 7.2)
		Physical workload	4 (1.7, 9.5)
		Low altitude	3.2(1.23, 8.31)
12	Demelash S et al. 2019 [35]	≤ once per week consumption of hem iron source foods	2.54 (1.53, 6.24)
		≤ once per week vegetable consumption	2.15 (1.48, 4.78)
		Maternal illiteracy	1.7 (1.01, 5.1)
		Low body mass index (BMI)	2.72 (1.92, 5.43)
		Drinking of tea	4.2 (2.37, 10.24)

*AOR* adjusted odds ratio, *CI* confidence interval

### The pooled magnitude of anemia among adolescent girls in Ethiopia

**Fig. 2:**
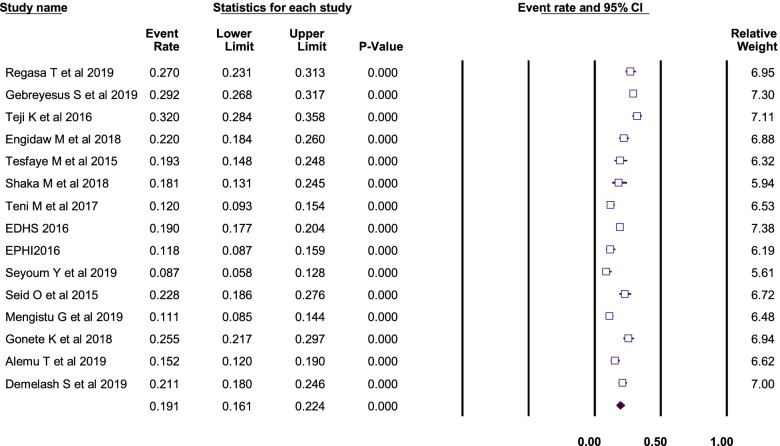
Forest plot for the pooled magnitude of anemia among adolescent girls in Ethiopia, 2021

Fifteen studies were included in this systematic review and meta-analysis to estimate the pool magnitude of anemia among adolescent girls in Ethiopia. Using the random-effects model, the pooled magnitude of anemia among adolescent girls in Ethiopia was 19.1% (95%CI: 16.1%, 24.6%). The heterogeneity among the studies used to estimate the pooled anemia among the adolescent girls of Ethiopia was high (I^2^ = 93.3%; *p*-value < 0.001) (Fig. 2).

**Fig. 3:**
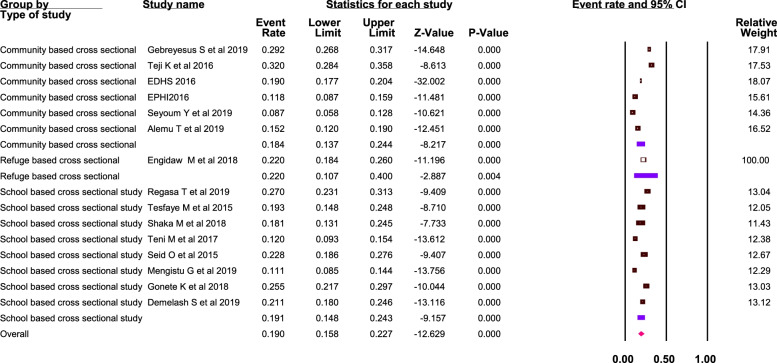
Forest plot for the subgroup analysis by study type, Ethiopia, 2021

Sub-group analysis by study type was performed and the magnitude of anemia was 19.1% among the school-based cross-sectional studies, 18.4% among community-based cross-sectional studies, and 22% in the refugee camp. The difference among these sub-groups was not statistically significant (Q-value = 0.22, *P*-value = 0.89) (Fig. 3).

Sensitivity analysis was performed for the effect size of anemia by removing data from the meta-analytic model to examine influence of studies on the overall pooled estimate. Accordingly, the sensitive analysis resulted in the magnitude of anemia 19.0% (95%CI: 15.8%, 25.2%) revealed no change on the overall pooled magnitude of anemia and heterogeneity remains statistically significant.

### Factors associated with anemia among adolescent girls in Ethiopia

**Fig. 4:**
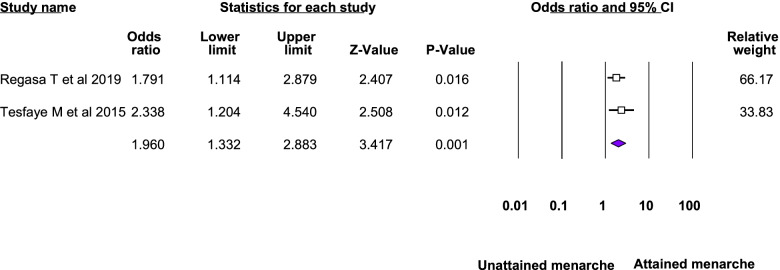
Forest plot for attained menarche, Ethiopia, 2021

**Fig. 5:**
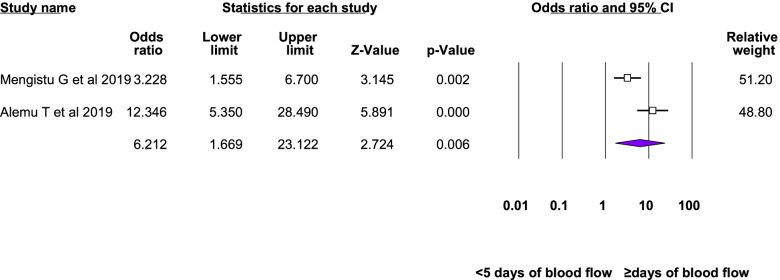
Forest plot for ≥ 5 days of blood flow during menses, Ethiopia, 2021

**Fig. 6:**
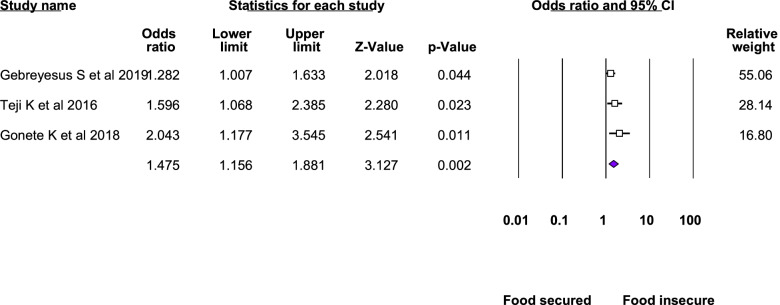
Forest plot for food insecurity, Ethiopia, 2021

**Fig. 7:**
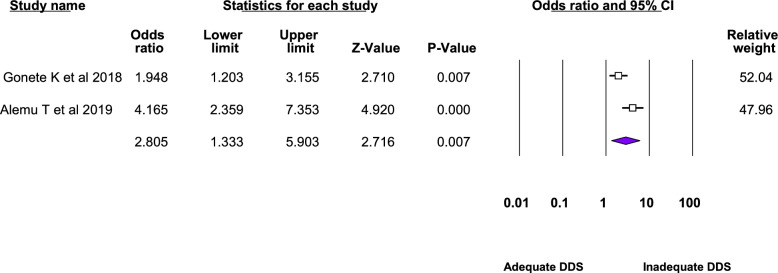
Forest plot for inadequate diet diversity score, Ethiopia, 2021

**Fig. 8:**
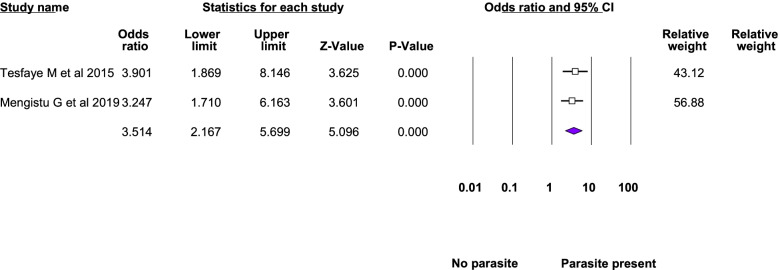
Forest plot for presence of intestinal parasite, Ethiopia, 2021

**Fig. 9:**
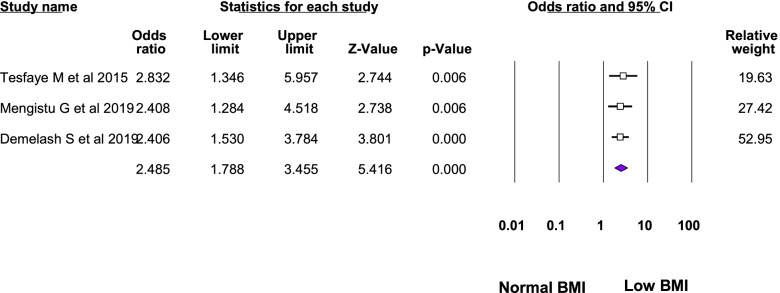
Forest plot for low BMI (< 18.5 kg/m.^2^), Ethiopia, 2021

**Fig. 10:**
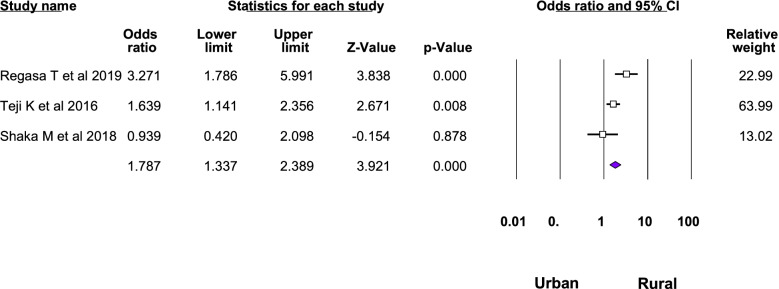
Forest plot for rural residence, Ethiopia, 2021

Twelve studies were included in the meta-analysis of the associated factors for anemia among adolescent girls [21–23, 25, 26, 28–31, 33–35]. A factor that was statistically significant in at least two studies was included in the meta-analysis. Six statistically significant factors were found in this meta-analysis: Attained menarche, ≥ 5 days of blood flow during menses, food insecurity, inadequate diet diversity score, presence of intestinal parasite, low body mass index, and rural residence. The pooled adjusted odds ratio ranged from 1.48 to 6.21. Heterogeneity was observed among the studies included in the analysis of food insecurity, rural residence, inadequate diet diversity score, and ≥ 5 days of blood flow during menses (I^2^: 25.33, 69.3, 74.96, and 82.17, respectively) (Table 3, Figs. 4, 5, 6, 7, 8, 9, 10).

**Table 3:** Summary of meta-analysis for the associated factors of anemia among Ethiopian adolescent girls, 2021

Associated factors	Number of studies	AOR (95%CI)	*P*-value	Heterogeneity			Egger’s test (*p*-value)
				Q-value	*P*-value	I^2^	
Attained menarche	2	1.96(1.33,2.88)	0.001	0.41	0.52	0.000	Not applicable *
≥ 5 days of blood flow during menses	2	6.21(1.67,23.12)	0.006	5.61	0.018	82.17	Not applicable *
Food insecurity	3	1.48(1.16, 1.88)	0.002	2.68	0.26	25.33	0.034
Inadequate diet diversity score	2	2.81(1.33,5.9)	0.007	0.99	0.046	74.96	Not applicable *
Presence of intestinal parasite	2	3.51(2.17,5.69)	< 0.001	0.14	0.71	0.000	Not applicable *
Low BMI	3	2.49(1.79,3.46)	< 0.001	0.15	0.93	0.000	0.43
Rural residence	3	1.79(1.34, 2.39)	0.06	6.51	0.04	69.3	0.97

*AOR* Adjusted Odds Ratio, *CI* confidence interval, *BMI* Body mass index

^*^at least three studies are required

### Publication bias

**Fig. 11:**
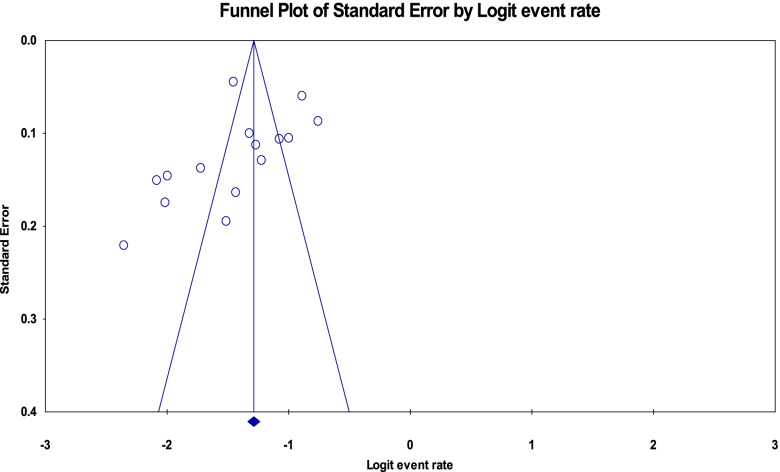
Funnel plot for publication bias of studies included to pooled magnitude of anemia among adolescent girls of Ethiopia, 2021

The funnel plot for the studies included to pooled the magnitude of anemia was tilted to the left side (asymmetric) which showed publication bias but Egger’s test was not statistically significant (*p*-value = 0.134) (Fig. 11).

**Fig. 12:**
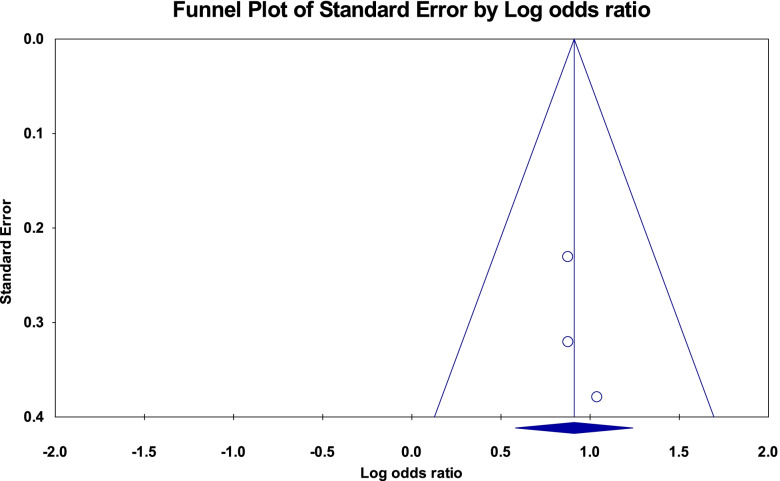
Funnel plot for publication bias of studies included for low body mass index, Ethiopia, 2021

**Fig. 13:**
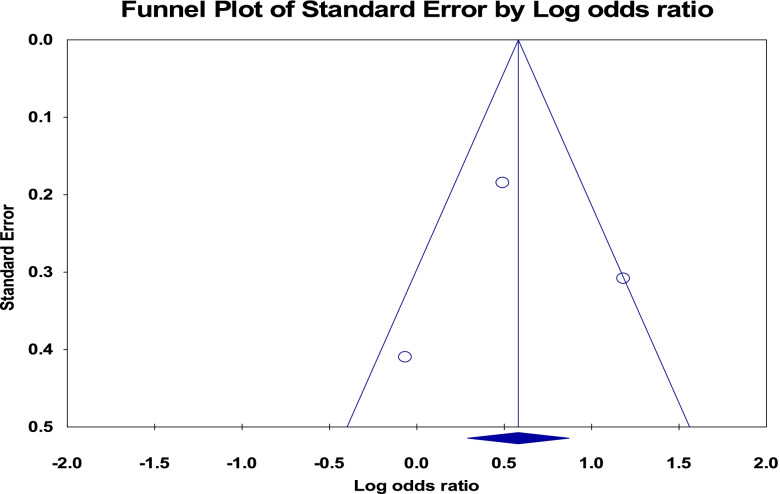
Funnel plot for publication bias of studies included for rural residence, Ethiopia, 2021

The funnel plots for the studies included to pooled the odds ratio for the low body mass index and rural residence were tilted to the right side (asymmetric) which showed publication bias but the Egger’s test was not statistically significant (*p*-value = 0.43 and 0.97, respectively) (Figs. 12 and 13).

**Fig. 14:**
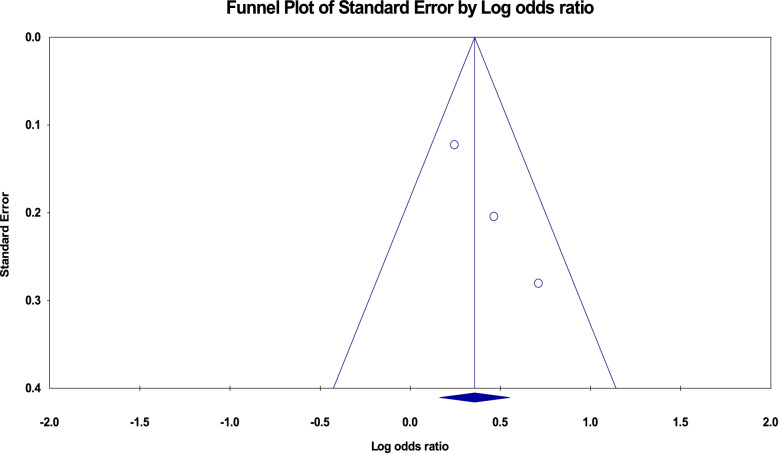
Funnel plot for publication bias of studies included for food insecurity, Ethiopia, 2021

The funnel plot was asymmetric (Fig. 14) and Egger’s test was statistically significant (*p*-value = 0.034) for the studies included to pooled the odds ratio of food insecurity which showed publication bias. Publication bias for attained menarche, inadequate diet diversity score, ≥ 5 days of blood flow during menses, and presence of intestinal parasite were not assessed due to a limited number of studies.

## Discussion

Generally, there were limited numbers of studies available in Ethiopia on the magnitude and associated factors of anemia among adolescent girls. In this meta-analysis, the random-effects model was used due to the significant heterogeneity among studies. We found that the pooled magnitude of anemia among adolescent girls in Ethiopia was 19.1% (95%CI: 16.1%, 24.6%). The pooled magnitude of anemia among adolescent girls of this meta-analysis was lower as compared to anemia among national representing adolescent girls in Nepal 31% [36], India 41.1% [37], (Indonesia 30%, SriLanka 40%, Bangladesh 40%, Myanmar 45.2%, and Pakistan 54%) [38] but higher than the magnitude of anemia among adolescent girls in Thailand 17% [39], and Turkey [40]. The differences in magnitude of anemia could be due to differences in economy, socio-culture, and dietary practices. The finding of this meta-analysis revealed that anemia among adolescent girls in Ethiopia was a mild public health problem. But it is important to consider that a mild public health problem of anemia among adolescent girls could have consequences especially for those adolescent girls who enter the reproductive age; risk of low birth, preterm delivery, perinatal mortality, and postpartum hemorrhage [39, 41]. Anemia in adolescent girls could increase morbidity, absenteeism, impaired cognition, and low school achievements [42].

In this systematic review and meta-analysis, associated factors for anemia among Ethiopian adolescent girls were identified. Adolescent girls who attained menarche were at higher risk of anemia. This finding was consistent with the finding of another study in India [43]. Menarche and physiological increase in hemoglobin level among adolescent girls can cause anemia [44]. Five days and above blood flow during menses was statistically significant factor for anemia among Ethiopian adolescent girls. The mean blood loss per menstrual period is 30 ml per cycle but a chronic loss of more than 80 ml is associated with anemia even though most females are unable to measure their blood loss. If the menstrual flow requires a change of menstrual products every 1–2 h, it is considered excessive [43, 45–47]. Another statistically significant factor for anemia among Ethiopian adolescent girls was food insecurity. Food insecurity could be associated with an inadequate intake of micronutrients like iron, vitamin B6, B9, and B12. These food insecure adolescents may consume fewer micronutrients from grain sources than did food-secure adolescents. Food-insecure adolescents are vulnerable to anemia due to fewer monetary and food resources [48, 49].

In this systematic review and meta-analysis, we found that the odds of anemic adolescent girls were higher among those with inadequate diet diversity score. A similar finding was found in studies conducted in Nepal [36], India [50], Nigeria [51], and resource-poor areas (Bangladesh, Burkina Faso, Mali, Mozambique, Philippines) [52]. Generally, there is poor diet quality among adolescent girls who live in low and middle-income countries including Ethiopia which may result in anemia and other micronutrient deficiencies [53, 54].

This systematic review and meta-analysis showed an association of intestinal parasite and anemia among Ethiopian adolescent girls. Supporting findings were found in studies conducted in Bangladesh [55], Brazil [56], Vietnam [57], and Tamil Nadu [58]. Intestinal parasite causes intestinal blood loss through feeding and oozing of blood at the attachment sites, iron deficiency, and protein deficiency resulting in anemia [55, 59]. Furthermore, intestinal parasite could interfere food intake, absorption, storage, and use of many nutrients such as iron, vitamin B_12_, folic acid, vitamin C, and vitamin A which contribute to anemia [39]. Our study further indicated that adolescent girls with low body mass index (BMI) were at higher risk of anemia. This is supported by other studies conducted in Iran [60], Greater Noida [61], and Nepal [62]. This is because adolescent girls with low body mass index are more likely to have micronutrient deficiencies which may resulted in anemia [60, 62]. Finally, our study revealed that adolescent girls who live in rural areas were at higher risk for anemia. A similar finding was found in studies conducted in India [63]. Adolescent girls in rural areas consume a poor-quality diet, and there is poor practice of personal hygiene and environmental sanitation that might contribute to the development of anemia [64].

This systematic review and meta-analysis has strengths like using of comprehensive search strategy with the involvement of more than two author reviewers in each step of the review process. The PRISMA 2020 guideline was followed during the reviewing process. But this systematic review and meta-analysis has a certain limitation which includes a small number of studies, all the regions and city administrations in Ethiopia were not represented by the primary studies included in this systematic review and meta-analysis. All the studies were cross-sectional studies which could affect the temporal relationship. The presence of high heterogeneity and significant publication bias (based on the Egger test) and few studies for meta-analysis of some factors are another limitation. Anemia level was not stratified due to the negligible percentage of mild, moderate, and severe anemia. Thus, caution is required in the interpretation and generalizing of these findings.

## Conclusion

The pooled magnitude of anemia among Ethiopian adolescent girls was 19.1% depicting a mild public health problem. The significant associated factors were attained menarche, ≥ 5 days of blood flow during menses, food insecurity, inadequate diet diversity score, presence of intestinal parasite, low body mass index, and rural residence. It is important to encourage consumption of diversified diets, control of intestinal parasite through regular deworming of adolescent girls in addition to improving personal hygiene and sanitation. It is also good to counsel adolescent girls concerning their menstrual health, and adolescent-specific therapies should be there for adolescent girls with long blood flow during menses after detailed evaluation. At state level, the issue of addressing health and nutrition wellness of adolescent girls should be center of concern in health, nutrition, agriculture, research, strategies and policies in Ethiopia believing that adolescents are the bridge of generations that connect childhood to adulthood in assuring sustainable development.

## Supplementary Information

**Additional file 1.** PRISMA 2020 Checklist.

## Data Availability

All data regarding this systematic review and meta-analysis are contained and presented in this systematic review and meta-analysis document.

## Abbreviations

AOR: Adjusted Odds Ratio
CI: Confidence Interval
CINAHL: Cumulative Index to Nursing and Allied Health Literature
DDS: Diet Diversity Score
EDHS: Ethiopian Demographic and Health Survey
PRISMA: Preferred Reporting Items for Systematic Review and Meta-Analysis
SNNP: South Nation, Nationalities and Peoples of Ethiopia
UNICEF: United Nation International Children’s Emergency Fund
WHO: World Health Organization
